# MRI in predicting the response of gastrointestinal stromal tumor to targeted therapy: a patient-based multi-parameter study

**DOI:** 10.1186/s12885-018-4606-0

**Published:** 2018-08-13

**Authors:** Lei Tang, Jian Li, Zi-Yu Li, Xiao-Ting Li, Ji-Fang Gong, Jia-Fu Ji, Ying-Shi Sun, Lin Shen

**Affiliations:** 1 Department of Radiology, Peking University Cancer Hospital & Institute, Key laboratory of Carcinogenesis and Translational Research (Ministry of Education), No. 52Fu Cheng Road, HaiDian District, Beijing, 100142 China; 2 Department of Gastroenterology, Peking University Cancer Hospital & Institute, Key laboratory of Carcinogenesis and Translational Research (Ministry of Education), No.52 Fu Cheng Road, HaiDian District, Beijing, 100142 China; 3 Department of Gastrointestinal Surgery, Peking University Cancer Hospital & Institute, Key laboratory of Carcinogenesis and Translational Research (Ministry of Education), No.52Fu Cheng Road, HaiDian District, Beijing, 100142 China

**Keywords:** Magnetic resonance imaging, Diffusion-weighted imaging, Apparent diffusion coefficient, Gastrointestinal stromal tumor, Response evaluation, Targeted therapy

## Abstract

**Background:**

To investigate the performance of quantitative indicators of MRI in early prediction of the response of gastrointestinal stromal tumor (GIST) to targeted therapy in a patient-based study.

**Methods:**

MRI examinations were performed on 62 patients with GIST using 1.5 T scanners before and at two and 12 weeks after treatment with targeted agents. The longest diameter (LD) and contrast-to-noise ratio (CNR) of the tumors were measured by T2-weighted imaging (T2WI), and the apparent diffusion coefficient (ADC) was determined using diffusion-weighted imaging (DWI). The pre-therapy and early percentage changes (%Δ) of the three parameters were compared with regard to their abilities to differentiate responder and non- responder patients, using ROC curves.

**Results:**

There were 42 patients in responder and 20 in non-responder group. After two weeks of therapy, the percentage changes in the ADC and LD were significantly different between the two groups (ADC: responder 30% vs. non- responder 1%, Z = − 4.819, *P* < 0.001; LD: responder − 7% vs. non- responder − 2%, Z = − 3.238, *P* = 0.001), but not in T2WI-CNR (responder − 3% vs. non-responder 9%, Z = − 0.663, *P* = 0.508). The AUCs on ROC for %ΔLD, %ΔT2WI-CNR and %ΔADC after two weeks of therapy were 0.756, 0.552 and 0.881, respectively, for response differentiation. When %ΔADC ≥15% was used to predict responder, the PPV was 93.3%.

**Conclusions:**

The percentage change of the ADC after two weeks of therapy outperformed T2WI-CNR and longest diameter in predicting the early response of GIST to targeted therapy.

## Background

Gastrointestinal stromal tumor (GIST) is the most common mesenchymal neoplasm that originates from the gastrointestinal tract [1]. The targeted agents of imatinib mesylate (Gleevec; Novartis, Basel, Switzerland) [1, 2] and sunitinib maleate (Sutent; Pfizer, New York, NY) [3] in the treatment of GIST had shown remarkable efficacy and been honoured as “rare paradigm” amongst modern anticancer therapies [2].

Predicting the efficacy of targeted agents for GIST at early stage is crucial for optimizing patient regimens and avoiding unnecessary systemic toxicity, expense and treatment delays [3]. Choi et al. [4] had proposed criteria that combined the size and CT values, which outperformed RECIST criteria in response evaluation of GIST. However, it usually takes 2–3 months’ time interval for CT to detect the response change, because of the radiation impairment and insensitive soft tissue contrast.

MRI is another commonly used modality that can provide both morphological and functional indicators. T2-weighted imaging (T2WI) can reflect the water content, through which to quantify the cystic or myxoid degeneration of GIST to targeted therapies [5]. Diffusion-weighted MRI (DWI) sensitively reflects the microscopic mobility of water molecules, through which pathological changes can be detected by calculating the apparent diffusion coefficient (ADC) [6, 7]. Previous lesion-based studies have revealed that the changes in the ADC after targeted therapy were associated with the response of GIST lesions [8–10]. To our knowledge, no patient-based study has been conducted to further compare the clinical performance of these variables. The purpose of this patient-based study was to compare the performances of various MRI indicators in early response prediction of GIST just after two weeks of initial therapy through the comparison with three months’ treatment outcome, and to propose thresholds for clinical practices.

## Materials and methods

### Patients

Our institutional review board approved this prospective MRI study. All of the included patients signed written informed consent form. The entry criteria for the patients were as follows: (1) unresectable or metastatic GIST or a primary lesion received neoadjuvant targeted therapy; (2) at least one solid lesion > 1 cm in diameter or cystic lesion with wall thickness > 1 cm; (3) imatinib (400 mg/day, PO) or sunitinib (50 mg/day, PO) single-drug targeted treatment. A total of 67 consecutive patients who met the entry criteria were scanned by MRI at three time points (pre-therapy and at two weeks and twelve weeks post-therapy).

The exclusion criteria were as follows: (1) contraindications for MRI examination (no patients were excluded according to this criterion); (2) discontinuation of MRI follow up during therapy (one patient was excluded); (3) severe complications arising from targeted agents that led to treatment interruption or dosage adjustment (two patients were excluded); (4) severe motion artifacts on respiratory-triggering T2WI and distortion or artifacts on DWI that influenced the stable measurement and comparison of the quantitative parameters (two patients were excluded).

Finally, 62 consecutive patients (41 men and 21 women; age range, 25–87 years; median age, 55 years; 22 patients with unresectable lesions, 34 patients with post-operative metastasis, 6 patients received neoadjuvant targeted therapy; 44 patients received imatinib treatment, and 18 patients received sunitinib treatment) met the above criteria and were enrolled in the study.

### MRI examination

The patients fasted overnight and were intramuscularly administered 20 mg anisodamine (Minsheng Pharmaceutical Group Company, Hangzhou, China) to inhibit gastrointestinal motility at 15 min prior to MR examinations. Pure water (800–1000 mL) was orally administered at 10 min after the above hypotonic procedure.

MR examinations were performed with a 1.5 T scanner. A full-range abdominal T2-weighted single-shot fast spin-echo sequence (SSFSE: TR/TE, 3000 ms/90 ms; matrix size, 384 × 256; section thickness, 5 mm; intersection gap, 1 mm; field of view, 360–400 mm; NEX, 0.57) on coronary plane was initially performed to detect and locate the target lesion. Then axial MR imaging was performed, including a T1-weighted dual fast spoiled gradient-recalled echo sequence (dual-FSPGR: TR/TE, 200/2.3 [out-of-phase], 200/4.6 [in-phase]; flip angle, 85°; matrix size, 320 × 160; section thickness, 5 mm; intersection gap, 1 mm; field of view, 360–400 mm; NEX, 1; breath holding) and T2-weighted fast-recovery fast spin-echo sequence (FRFSE: TR/TE, 2 respiratory intervals/85 ms; matrix size, 320 × 224; section thickness, 5 mm; intersection gap, 1 mm; field of view, 360–400 mm; NEX, 2; respiratory triggering).

A single-shot echo-planar DWI sequence (TR/TE, 2750 ms/min; matrix size, 128 × 128; section thickness, 5 mm; intersection gap, 1 mm; field of view, 360–400 mm; NEX, 4) was performed to cover the whole target lesion. Motion-probing gradients (MPGs) were applied in three orthogonal directions (*x*-, *y*- and *z*-axes), and the *b*-factors were 0 and 1000 s/mm^2^. It was carried out with segmented breath holding if the imaging time exceeded the patients’ breath-holding endurance [11].

### Image analysis and therapeutic response assessment

All image data were transferred to a commercially available workstation (AW4.2; GE Medical Systems, Milwaukee, WI, USA). MR images obtained at all three time points were analysed in consensus by two experienced radiologists who were blinded to the therapeutic response results, to choose the target lesions and determine the ROI placement.

Target lesions were determined according to Choi criteria [4]. When multiple lesions were detected in one patient, a maximum of five largest lesions were identified as target lesions. The manually contoured region of interest (ROI) around the lesion border on the ADC map and T2WI was assessed on the slices where the largest lesion was located. The corresponding mean ADC was derived from the following formula: ADC = [ln (S2/S1)] / (*b*1-*b*2) (S1 and S2 represent the signal intensities of the lesions from *b*1 = 1000 s/mm^2^ and *b*2 = 0 s/mm^2^, respectively). The CNR on T2WI was calculated with the following formula: CNR = (S_GIST_-S_muscle_)/ Sd_background_ (S_GIST_ and S_muscle_ represent the signal intensities of the GIST lesions and the psoas muscle, respectively, and Sd denotes the standard deviation). When large areas of cystic or myxoid degeneration signal (signal as free water on T1WI and T2WI images, with sharp boarder to nearby solid tissues) were detected on the pre-therapy images, they were carefully excluded from the ROIs at all three time points.

The percentage changes in the quantitative parameters after two weeks of therapy were calculated with the following formulas: %ΔParameter = (Parameter_post_ - Parameter_pre_) / parameter_pre_ × 100%. Therapeutic response was determined by the situation of the tumors after three months’ targeted therapy. Because it’s hard to perform CT and MRI simultaneously, especially to perform CT in just two weeks post initial therapies, so the modified Choi criteria of MRI version was adopted [4, 9]: the patients were classified as responder if the target lesion exhibited a 10% or greater reduction in the LD or displayed apparent cystic or myxoid degeneration on T2WI after three months’ therapy; otherwise, they were considered as non- responder.

### Statistical analysis

All statistical analyses were performed with SPSS software program (SPSS for Windows, Ver. 16.0; SPSS, Inc., Chicago, IL) and STATA 11.0. The pre-therapy LD, T2WI-CNR and ADC and their percentage changes after treatment of the target lesions between the responder and non-responder groups were compared with Student’s t-test (if normally distributed) or the Mann-Whitney test (if non-normally distributed). Repeated measures Analysis of Variance was conducted to compare the variation trend of radiological parameters between responder and non-responder. Bonferroni correction was used for multiple comparisons. Receiver operating characteristic (ROC) curves were generated to compare the performances of the three parameters and their early changes. Youden’s J-statistic was used to calculate cut-points from ROC curves. The positive predictive value (PPV) and negative predictive value (NPV) in the prediction of responder and non-responder were obtained. Statistical significance was declared at *P* < 0.05.

## Results

### Common results

One hundred and forty-one target lesions were identified on pre-therapy T2WI and DWI images, and the LD, T2WI-CNR and ADC could be reliably measured and traced at all three examination time points. There were 27 primary GIST lesions (cardia-fundus, 5; gastric body, 10; gastric antrum, 4; duodenum, 5; jejunum, 2; rectum, 1) and 114 metastatic lesions (mesentery/peritoneum, 56; liver, 57; spleen, 1).

According to the modified Choi criteria, 42 patients showed responder and 20 non-responder to the targeted therapy. No age or gender difference was found between two groups (*P* > 0.05).

### Pre-therapy and variation trend of quantitative parameters in differentiation of the responses

All of the pre-therapy quantitative parameters were normally distributed (Kolmogorov-Smirnov test, ADC: *Z* = 0.760, *P* = 0.610; LD: *Z* = 1.165, *P* = 0.133; T2WI-CNR: *Z* = 0.932, *P* = 0.350).

No significant differences in the three baseline parameters were found between the responder and non- responder groups (ADC: 1.18 ± 0.27× 10^− 3^ mm^2^/s vs. 1.17 ± 0.31× 10^− 3^ mm^2^/s, *F* = 0.001, *P* = 0.972; LD: 70.58 ± 33.40 mm vs. 59.16 ± 40.55 mm, *F* = 0.914, *P* = 0.343; T2WI-CNR: 39.28 ± 22.62 vs. 30.32 ± 18.19, *F* = 0.359, *P* = 0.551) (Table 1).

**Table 1:** Quantitative parameters between the responder and non-responder groups

Time point	Responder	Non-responder	*F*	*P*
ADC (mm^2^/s)			19.378	*< 0.001*
Baseline	1.18 ± 0.27	1.17 ± 0.31	0.001	0.972
Week 2	1.60 ± 0.40	1.19 ± 0.38	13.620	*< 0.001*
Week 12	1.99 ± 0.56	1.20 ± 0.31	33.293	*< 0.001*
LD (mm)			0.15	0.700
Baseline	70.58 ± 33.40	59.16 ± 40.55	0.914	0.343
Week 2	63.51 ± 29.79	58.98 ± 38.33	0.125	0.725
Week 12	48.07 ± 27.86	71.57 ± 46.37	6.044	*0.017*
T2WI-CNR			0.359	0.551
Baseline	39.28 ± 22.62	30.32 ± 18.19	1.930	0.170
Week 2	41.56 ± 24.22	37.43 ± 24.21	0.920	0.341
Week 12	36.29 ± 23.41	40.82 ± 28.27	0.429	0.515
%ΔADC = (ADC_post_ - ADC_pre_) / ADC_pre_			*t/Z*	*P*
Week 2	0.30 (−0.03, 1.38)	0.01(−0.29, 0.30)	− 4.819	*< 0.001*
Week 12	0.67 (−0.07, 3.13)	0.06 (− 0.26, 0.52)	−4.890	*< 0.001*
%ΔSize = (LD_post_ - LD_pre_) / LD_pre_			*t/Z*	*P*
Week 2	−0.07 (− 0.36, 0.07)	−0.02 (− 0.12, 0.56)	−3.238	*0.001*
Week 12	−0.28 (− 0.91, 0.08)	0.13 (− 0.07, 0.85)	− 6.120	*< 0.001*
%ΔCNR = (CNR_post_ - CNR_pre_) / CNR_pre_			*t/Z*	*P*
Week 2	−0.03 (− 0.57, 2.11)	0.09 (− 0.36, 4.56)	−0.663	0.508
Week 12	−0.04 (−1.10, 7.43)	0.28 (− 0.70, 2.88)	− 1.962	*0.050*

Comparison of the tumor ADC, LD and T2WI-CNR at three time points (mean ± sd) and the percentage change at two and twelve weeks after therapy (median and range) between the responder and non-responder groups

Repeated measures Analysis of Variance showed responder and non-responder demonstrated significantly different variation trend in ADC (*P* < 0.001), and responder and non-responder persistently demonstrated different ADC values at week 2 and week 12. In contrast, the overall variation trends of LD and CNR were similar between responder and non-responder (*P* = 0.700 and 0.551, respectively), although responder and non-responder demonstrated significantly different LD values at week12.

**Fig. 1:**
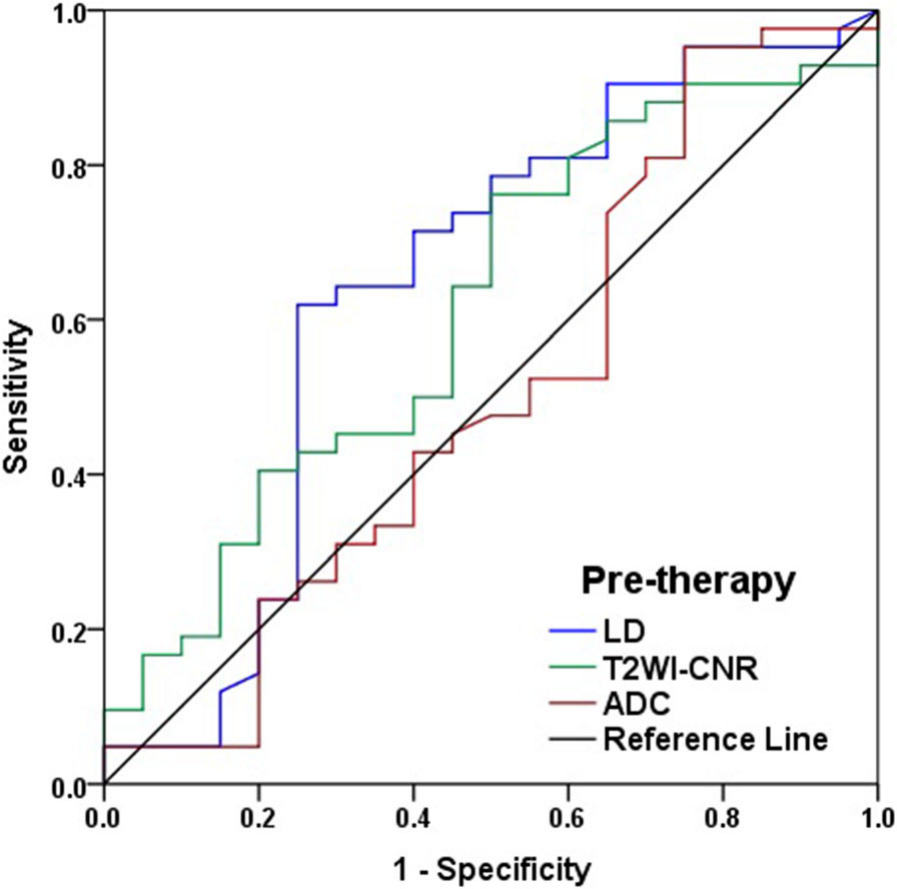
The efficacies of pre-therapy quantitative parameters with regard to their capacities for response prediction. The areas under the curve (AUCs) for pre-therapy longest diameter (LD), T2-weighted imaging contrast-to-noise ratio (T2WI-CNR) and apparent diffusion coefficient (ADC) were 0.644, 0.615 and 0.508, respectively

According to the ROC curves, the AUCs for pre-therapy LD, T2WI-CNR and ADC were 0.644 (95% CI, 0.479 to 0.809), 0.615 (95% CI, 0.462 to 0.768) and 0.508 (95% CI, 0.337 to 0.675), respectively, for response differentiation (Fig. 1). There was no statistical difference of AUCs between pre-therapy ADC and other two indicators (ADC vs. LD, Bonferroni adjusted *P* = 0.411; ADC vs. T2WI-CNR, Bonferroni adjusted *P* = 0.593).

### Response prediction using the percentage changes (%Δ) of the parameters

None of the parameter was normally distributed after two weeks of therapy (Kolmogorov-Smirnov test, %ΔADC: *Z* = 0.147, *P* = 0.002; LD: *Z* = 0.117, *P* = 0.036; T2WI-CNR: *Z* = 0.214, *P* < 0.001).

The %ΔADC significantly differed between the two groups after two weeks of therapy (median, responder 30% vs. non-responder 1%, Z = − 4.819, *P* < 0.001). The %ΔLD between the two groups had statistical significance (median, responder − 7% vs. non-responder − 2%, Z = − 3.238, *P* = 0.001) but with only slightly difference. No significant difference was observed in the %ΔT2WI-CNR between the two groups (median, responder − 3% vs. non-responder 9%, Z = − 0.663, *P* = 0.508) (Table 1).

**Fig. 2:**
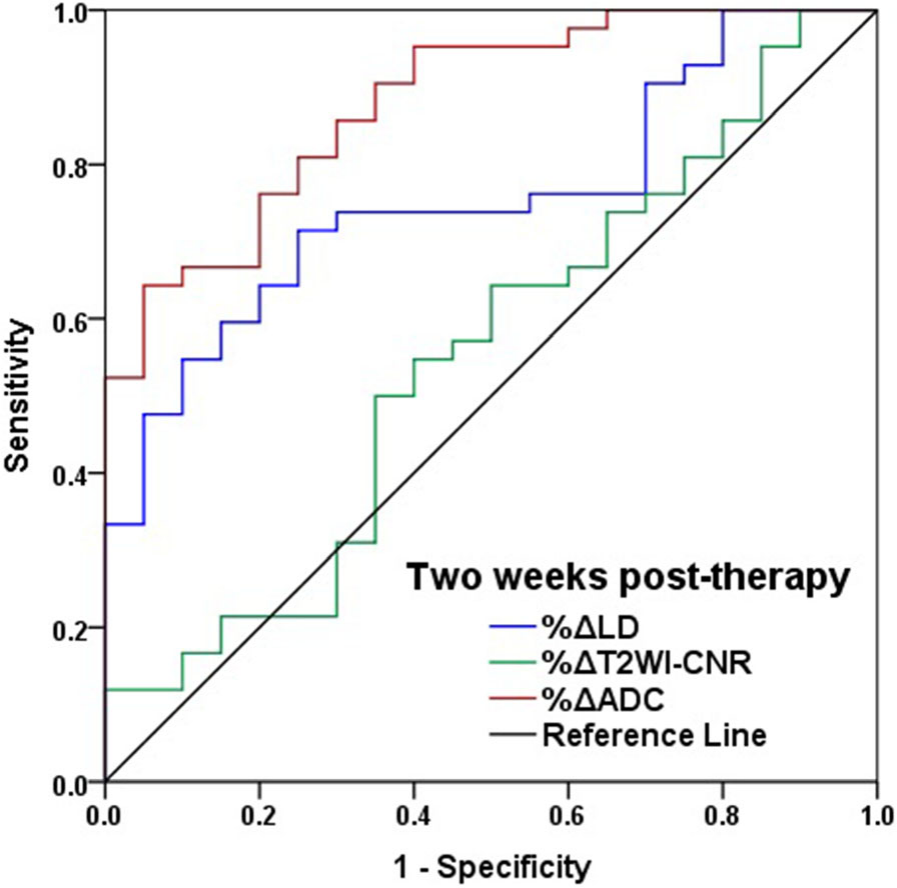
The efficacies of the percentage changes in the quantitative parameters for response prediction. The AUCs for the percentage increases in the LD, T2WI-CNR and ADC after two weeks of therapy were 0.756, 0.552 and 0.881, respectively

According to ROC curves, the AUCs for %ΔADC, %ΔLD and %ΔT2WI-CNR after two weeks of therapy were 0.881 (95% CI, 0.795 to 0.965), 0.756 (95% CI, 0.629 to 0.873) and 0.552 (95% CI, 0.395 to 0.711), respectively, for response differentiation (Fig. 2). There were statistical differences of AUCs between pre-therapy ADC and other two indicators (%ΔADC vs. %ΔLD, Bonferroni adjusted *P* < 0.001; %ΔADC vs. %ΔT2WI-CNR, Bonferroni adjusted *P* < 0.001). When %ΔADC ≥10% was used as a cut-off value to predict responder, the sensitivity was 0.762 and the specificity was 0.800, and when %ΔLD ≤ − 2% was used as a cut-off value, the sensitivity was 0.738 and the specificity was 0.700.

**Fig. 3:**
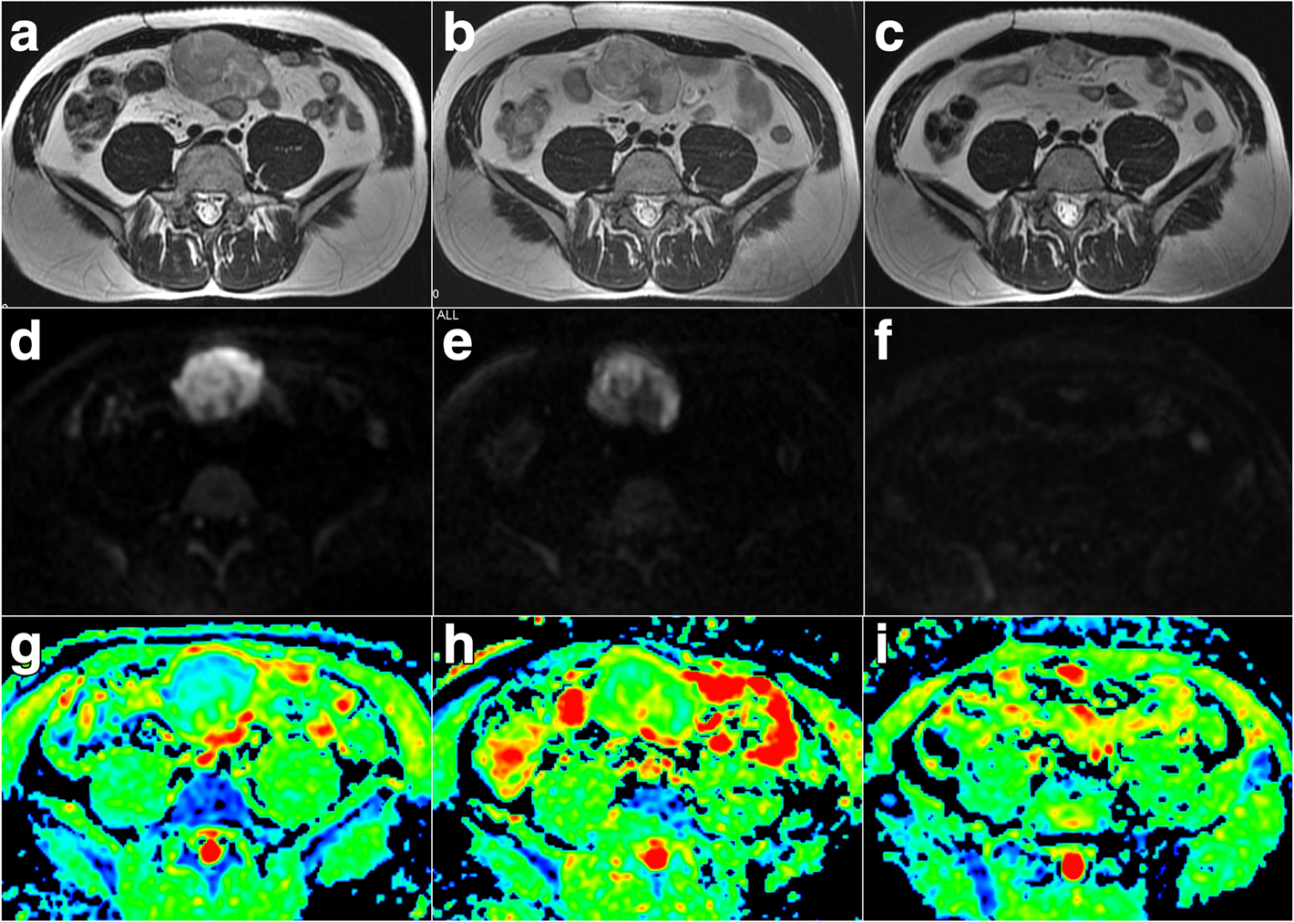
Pre-therapy, two-week and 12-week post-therapy images for a 43-year-old male patient from the responder group with abdominal metastatic gastrointestinal stromal tumor (GIST) lesions treated with imatinib mesylate. **a-c** Axial fast spin-echo T2-weighted MR images at three time points. The maximum tumor diameters were 7.5 cm before therapy (**a**), 7.2 cm at two weeks post-therapy (**b**) and 3.6 cm at 12 weeks post-therapy (**c**). The tumor-to-muscle CNRs were 47 before therapy (**a**), 43 at two weeks post-therapy (**b**) and 55 at 12 weeks post-therapy (**c**). **d-f** Axial diffusion-weighted MR images (DWI) with *b* = 1000 s/mm^2^ at three time points. The tumors ADCs were 0.86 × 10^− 3^ mm^2^/s before therapy (**g**), 1.36 × 10^− 3^ mm^2^/s at two weeks post-therapy (**h**) and 2.43 × 10^− 3^ mm^2^/s at 12 weeks post-therapy (**i**). The tumor ADC was significantly increased at two weeks after the targeted therapy (%ΔADC = 58.1%), whereas the changes in tumor size and the T2WI-CNR were not obvious (%ΔLD = − 4.0%, %ΔCNR = − 8.5%)

**Fig. 4:**
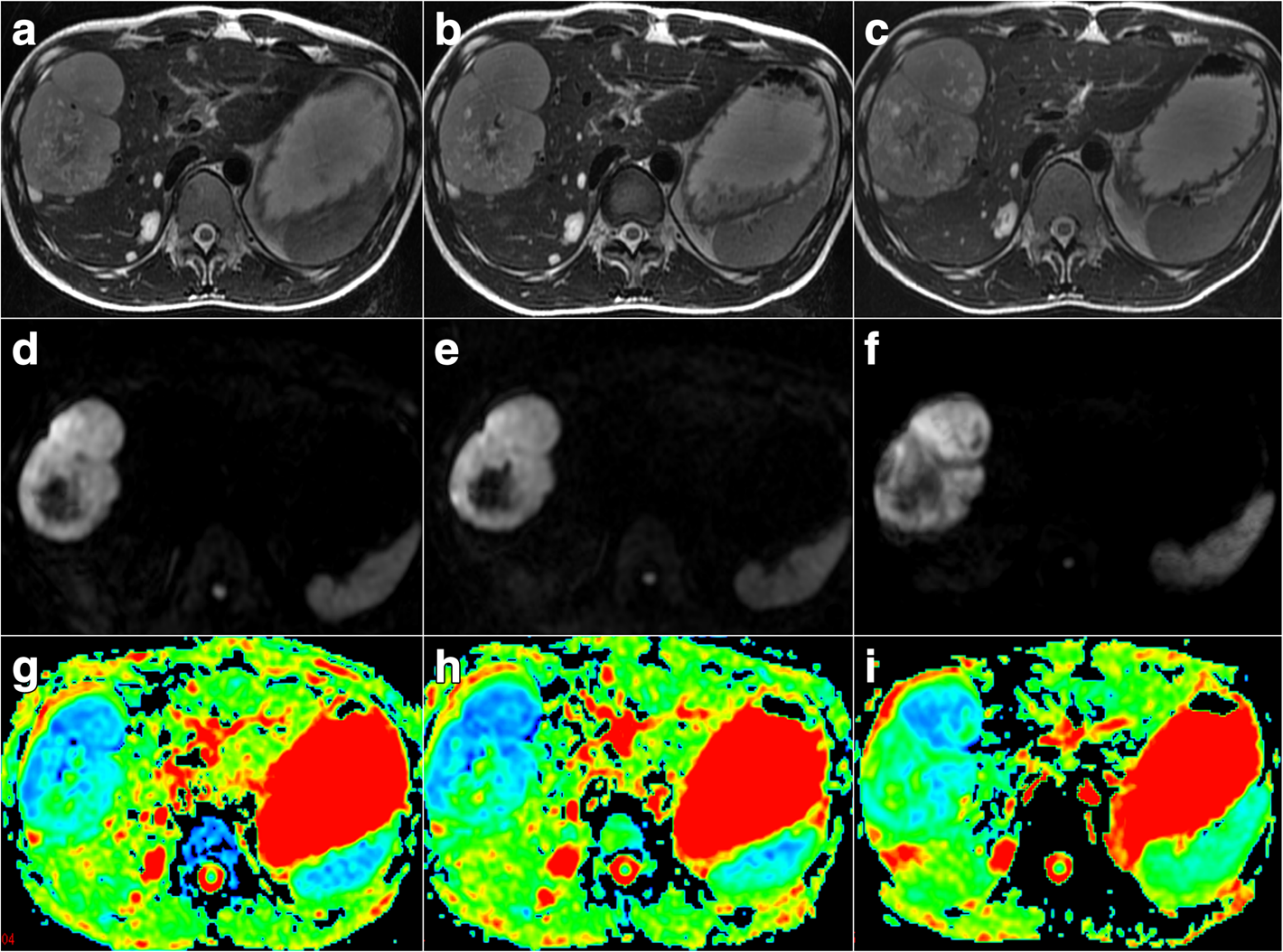
Pre-therapy, two-week and 12-week post-therapy images for a 31-year-old male patient from the non-responder group with hepatic metastasis treated with imatinib mesylate. **a-c** Axial fast spin-echo T2-weighted MR images at three time points. The maximum tumor diameters were 8.9 cm before therapy (**a**), 9.0 cm at two weeks post-therapy (**b**) and 9.8 cm at 12 weeks post-therapy (**c**). The tumor-to-muscle CNRs were 19 before therapy (**a**), 18 at two weeks post-therapy (**b**) and 20 at 12 weeks post-therapy (**c**). **d-f** Axial diffusion-weighted MR images (DWI) with *b* = 1000 s/mm^2^ at three time points. The tumor ADCs were 0.74 × 10^− 3^ mm^2^/s before therapy (**g**), 0.73 × 10^− 3^ mm^2^/s at two weeks post-therapy (**h**) and 0.89 × 10^− 3^ mm^2^/s at 12 weeks post-therapy (**i**). The tumor ADCs did not significantly change during the monitoring period. None of the three quantitative parameters displayed obvious changes at two weeks after the targeted therapy (%ΔADC = − 1.4%, %ΔLD = 1.1% and %ΔCNR = − 5.3%)

When %ΔADC ≥15% was used to predict responder, the PPV was 93.3% (28/30) and the NPV was 56.3% (18/32) (Fig. 3). When %ΔADC ≤1% was used to predict non-responder, the PPV was 85.7% (12/14) and the NPV was 83.3% (40/48) (Fig. 4).

## Discussion

Recent studies have confirmed the potential value of DWI in predicting the responses of malignant tumors to anticancer therapies [12, 13]. ADC changes may occur just weeks or even days after initial therapies. Furthermore, some literatures have determined that the ADC is more sensitive than size on lesion-based studies about response prediction of GIST [8–10]. It has been postulated that targeted therapy interferes with the metabolism of GIST cells, subsequently inducing apoptosis and shrinkage of tumor cells. The decrease in cell density liberates space for the Brownian motion of water molecules, alleviates the restriction of water mobility and consequently leads to an increase of ADC [9].

In our study, the AUC for the percentage change of ADC after two weeks of targeted therapy reached 0.881, which was higher than those for both the longest diameter and T2WI-CNR, in the performance of response prediction. Additionally, a 10% cut-off value was sufficient to attenuate measurement errors [14]. In contrast, although the percentage changes in the longest diameter between the two groups were statistically significant, the AUC was only 0.756 and the cut-off value was only 2% (means only 2 mm change in a 10 cm tumor), of which too small change might be easily offset by measurement errors and is not sufficient for clinical practices.

To our knowledge, this study is the first patient-based research investigating the prediction of GIST response to targeted therapy using DWI, and the resulted threshold has better clinical applicability than those of the previous lesion-based studies. After all, the determination of treatment regimens was patient-based, not lesion-based; and to GIST patients who often have multiple lesions, the lesion-based results may not applicable, since different lesions in same patient may have opposite change tendency. Based on above object, we investigated the practical threshold of the %ΔADC to provide additional information for clinical decision. The increase of %ΔADC ≥15% after two weeks of therapy indicated the likelihood of responder. This finding suggests that these patients could continue with their initial treatment. On the contrary, if the ADC showed no explicit increase after two weeks of therapy (%ΔADC ≤1%), then non-responder was highly suspected; however, considering that there was a 15% false-positive rate, shortening of the subsequent follow-up time intervals is suggested to further confirm the findings and facilitate the detection of early progression.

A previous study has reported the value of CNR on T2WI in the assessment of GIST response [5]. Theoretically, the responder of GIST to targeted therapy is often accompanied by cystic or myxoid degeneration, which demonstrates high signal on T2WI images and therefore increases the CNR. Stroszczynski et al. [5] have employed the T2WI-CNR as an indicator for evaluating the response of GIST to targeted agent and have found that the changes after two months of therapy differed between the responder and non-responder groups.

Interestingly, no difference was observed in %ΔT2WI-CNR between the responder and non-responder in our study. Two hypotheses may explain these inconsistent results. First, the T2WI-CNR may be not as sensitive as ADC in demonstrating early histopathological changes of GIST (Fig. 3). The findings of a study conducted by Huang et al. [15], in which they evaluated the chemotherapy response of non-Hodgkin’s lymphoma xenografts, appear to support this hypothesis. In their study, ADC increased as early as one week after initial therapy. However, a significant change of T2 value was observed only after two chemotherapy cycles. The parameter that reflects water molecule motion prevailed over water content. Second, intra-tumoral haemorrhage, which often signifies a responder [1], may confuse signal changes with a low T2WI signal, which hinders the increased signal of hypocellular degeneration to targeted therapy. The above effects partly explained the insensitivity of the T2WI-CNR to early responses.

There were several potential limitations of our study. First, the lowest *b* value possible is only 0 s/mm^2^ on our machine, and the ADC value may be influenced by microscopic perfusion. The use of low *b* values of 200–400 s/mm^2^ with the IVIM model and FROC model [16–18] would be more sensitive to diffusion. Second, we could not use the contrast-enhanced sequence repeatedly over a short time period because of ethical reasons, which may have introduced bias in the judgement of cystic or myxoid degeneration only by T1WI and T2WI. Third, comparison studies that involve histopathological assessments would be helpful to obtain better understanding of the correlation between the ADC and tumor cellularity, although histopathological validation by biopsy or surgery is not feasible for every lesion for obvious ethical and technical reasons. Last, PET/CT is the standard of care for GIST response assessment, but most of the patients could not be performed PET examinations because of economic factors, so we chose modified Choi criteria on MRI as standard, which was weaker than the prior one.

## Conclusions

In conclusion, the percentage change in the ADC of GIST after two weeks of targeted therapy exhibited a reliable performance in response prediction, which outperformed the T2WI-CNR and longest diameter. We suggest that patients continue with their treatment regimen if the percentage increase of the ADC is larger than 15% after two weeks of therapy. In contrast, if the ADC decreases or exhibits almost no change, a shortening of the follow-up time interval is recommended to detect possible drug resistance at an early stage.

## Abbreviations

ADC: Apparent diffusion coefficient
CNR: Contrast-to-noise ratio
DWI: Diffusion-weighted imaging
GIST: Gastrointestinal stromal tumor
LD: Longest diameter
T2WI: T2-weighted imaging
